# “Hyperkinetic” and “Hypokinetic”: Is There a Need for a Third Category (i.e., “Mixed”)?

**DOI:** 10.5334/tohm.997

**Published:** 2025-05-13

**Authors:** Sara M. Schaefer, Elan D. Louis

**Affiliations:** 1 Department of Neurology, Yale School of Medicine, New Haven, CT, USA; 2 Department of Neurology, University of Texas Southwestern Medical Center, Dallas, TX, USA; 3 Peter O’Donnell Jr. Brain Institute, University of Texas Southwestern Medical Center, Dallas, TX, USA

**Keywords:** Movement disorders, classification, clinical, nomenclature

## Abstract

Terminology in the field of movement disorders has evolved multiple times over the years. Traditionally, classification schema have utilized a two-category approach, with a hyper- and hypokinetic branchpoint as the first step towards describing movement disorders and elucidating phenomenological diagnoses. However, this terminology falls short, as it does not adequately capture the complexity of several abnormalities of movement- including ataxia and rigidity- at the electrophysiologic and phenotypic levels. Rather, these movement disorders are characterized by mixed hyper- and hypokinetic phenomena. We propose a third category, “mixed”, which would optimally classify the full range of movements.

## History of Movement Disorders Terminology – A Tale of Dualities

The terminology that relates to movement disorders seems to have been in a state of flux since its beginnings. As a relative late-comer to this mix, the term *movement disorders* itself did not even arise until coined by Stanley Fahn M.D. in the mid-20^th^ century [1]. Historically, the field of movement disorders was considered to encompass the study and treatment of disorders of the *extrapyramidal system*, a term that originated in the final two years of the nineteenth century in the setting of experimental physiology [2]. The initial coining of the term *extrapyramidal* occurred before there was a detailed neuroanatomical understanding of the structures involved; however, there was a growing scientific understanding that motor control did not only involve the corticospinal tracts. The extrapyramidal system was later defined as including the nuclei of the basal ganglia, which are deeply involved in motor control, in contrast to the pyramidal, or corticospinal, motor system, named for the “pyramids” that are seen anatomically in the brainstem. Underlying the terminology was the concept of duality of motor control: there was the pyramidal system, and the *extra*pyramidal system.

Although initially a useful construct, the term *extrapyramidal* has since fallen out of use for a multitude of reasons including its lack of pathophysiological or anatomical specificity. Many movement disorders originate from outside of the basal ganglia, involve other descending tracts, and/or relate to intimate connections between the “pyramidal” and “extrapyramidal” tracts [1]. The term “offers no insight into the nature or severity of the movement disorder” nor does it inform treatment [3]. Further work attempted to classify movement disorders based on anatomical localization of pathology [1], a strategy that was ultimately usurped by a reliance on the clinical phenotype (i.e., the quality of the movements themselves).

Phenotypically, disorders involving motor systems outside of the pyramidal tracts were defined as embracing one or more of the following features: 1) abnormal involuntary movements, 2) changes in tone of skeletal muscles with an increase or decrease in resistance to passive motions, 3) poverty or excess of movement, and 4) alteration of automatic movements [4]. On a more elemental level, these movements could be simply dichotomized into excess of movement (*hyperkinetic*) and poverty/paucity of movement (*hypokinetic*). This essential branchpoint has persisted reliably into modern classification schemes of, definitions of, and teaching tools for movement disorders [5,6].

Herein we will discuss the hyper- versus hypokinetic duality as well as challenges to this dichotomous scheme.

## Hyper- vs. Hypokinetic

When assessing the classification of abnormal movements as either hyperkinetic or hypokinetic, one should consider that this division may occur because of: 1) what is happening at the muscular level (“Electrophysiology classification”), or 2) the outward appearance or *result* of what is happening at the muscular level (“Phenotype classification”). Indeed, both electrophysiological and phenotypic aspects are incorporated into some phenomenological classification criteria such as the recent consensus criteria for myoclonus [7]. When one looks at how various abnormal movements are classified across educational resources, it is not always clear which of these two aspects (or whether both) has led to its classification as a hyper- or hypokinetic movement disorder. Many abnormal movements, including bradykinesia and tremor, fit cleanly into a hypokinetic or hyperkinetic category because both aspects (electrophysiological and phenotypic) appear to be distinctly hypo- or hyperkinetic. However, it is challenging to apply the hyper- or hypokinetic terminology to some phenomenologies. It should be noted that while disease states such as Parkinson’s disease and Huntington’s disease are well-known to feature both hyper- and hypokinetic phenomenologies, the discussion herein explores individual phenomenologies and their physiological and phenotypic discrepancies rather than syndromic disorders of the nervous system.

## Ataxia – A Challenge to the Dichotomous Scheme

Ataxia is difficult to characterize as either hyper- or hypokinetic. Although it is sometimes characterized under the hyperkinetic umbrella [5], it is commonly described in a way that avoids these terms.

The term “ataxia” encompasses the incoordination of rate, range, direction, force, timing, or organization of a movement [8], with the common pathophysiology being cerebellar pathway dysfunction. Limb ataxia (dysmetria and dysdiadochokinesia) relates mostly to the range and timing of a movement and may be described as an overshoot/undershoot when approaching a target. Dysmetria can appear in the arms as if the patient is attempting to put two negative ends of a magnet together; this manifests outwardly as superfluous movement to reach a target. Therefore, the outward appearance or phenotype of ataxia is hyperkinetic.

Similar to truncal and appendicular ataxia, ataxic speech demonstrates multiple phenotypic characteristics that may be considered hyperkinetic (prolonged phonemes or consonant sounds, perception of “vocal instability” or “vocal tremor”), hypokinetic (slow speech rate, monopitch, breathiness), or simply incoordinated [9]. The lack of articulatory precision in ataxic speech is described similarly to the above description of limb ataxia as “reduced control of range, velocity, force and timing of the articulators” [9].

Ataxia has been studied neurophysiologically with surface electromyography (EMG) during simple motor tasks, with some patients with cerebellar dysfunction demonstrating prolonged agonist and/or antagonist muscle activity interrupting the coordinated alternating pattern of agonist and antagonist activation as a target is approached, or pathologically overlapping agonist/antagonist muscle activation when a rapid alternating task is performed [8]. Interestingly, in a 1975 study, abnormal hyperactivation of either agonist or antagonist muscles (or both) was seen in both undershoot and overshoot. Thus, the authors concluded that cerebellar dysfunction may be considered a “failure in termination of muscular activity”, which would characterize ataxia as a “hyperkinetic” disorder at the muscular level [8].

Further studies confirmed this excessive duration of muscle bursts but also documented reduced peak force with slower and prolonged acceleration of the activity [10]. One potential explanation put forth was a reduction in phasic muscle force due to cerebellar dysfunction, and indeed reduced phasic signal has been demonstrated in animal models of cerebellar injury. A potential reduction of tonic motor drive due to cerebellar lesions might explain another common feature in cerebellar dysfunction, hypotonia [11].

Therefore, ataxia granularly represents a mix of excitatory and inhibitory forces. There is prolonged and inappropriately concurrent muscle activity, along with reduced peak force, slower but also prolonged muscle burst acceleration, and reduced tonic motor input. The system is uncoordinated, and also, it is not exclusively hyper- or hypokinetic.

## Rigidity- Another Challenge to the Dichotomous Scheme

As is the case with ataxia, rigidity is also difficult to characterize as either hyper- or hypokinetic. At the muscular level, rigidity is related to *hyper*activity of the muscles that is present with or without passive activation of the affected body part; on electromyography (EMG) studies, patients with Parkinson’s disease (PD) demonstrate higher EMG activity at rest, after voluntary activation with prolonged latency to a resting state, and during passive stretch [12,13]. The outward appearance of rigidity is a paucity of movement but this is mixed with increased muscle tone that is felt during passive range of motion on examination; the electrophysiological pathology is therefore decidedly hyperkinetic while the phenotype is mixed. Yet, rigidity is often characterized as a hypokinetic phenomenon, often paired with bradykinesia in the setting of parkinsonism.

## Is it Time for the Next Phase of Lexical Reorganization?

We have explored how the categorization of abnormalities of movement into hyper- versus hypokinetic branches as a first step toward phenomenological diagnosis is useful, but in some cases such as ataxia and rigidity, is problematic.

From an educational perspective, one may make the argument that a single common branchpoint is a simple step that initiates an easy framework for those learning movement disorders. Educationally, learners find simple algorithms to be more accessible when studying new material [14], but it may be more confusing if categorizations are made for unclear reasons or the reality is stretched too broadly for the sake of simplicity.

One thing we have learned over more than a century of study of the human brain and its relationship to movement is that concepts of duality do not often serve disorders of the central nervous system. For example, the terms “pyramidal” and “extrapyramidal” have been abandoned as poor representations of the complex reality of the interconnected anatomical and physiological systems that elicit movement. As another example, some resources have already abandoned the “kinesis” branchpoint in their organizational structure, instead clustering disorders based on a variety of phenotypic similarities (rhythmicity, pattern, or voluntariness) [15].

## A New Scheme

Perhaps instead of contorting to subdivide movement disorders into “hyperkinetic” or “hypokinetic” buckets, we should embrace the complexity of the system and retire the buckets altogether. Alternatively, it may be time to remove the constraints of a simple duality and add additional categories. In its simplest form, the terminology could change to “*Hyperkinetic*,” “*Hypokinetic*” and “*Mixed*,” which is our recommendation. This would avoid the need to contort and would be less confusing to learners, who are trying to understand rather than over-simplify.
